# Effects of Oral Administration of Fucoidan Extracted from *Cladosiphon okamuranus* on Tumor Growth and Survival Time in a Tumor-Bearing Mouse Model

**DOI:** 10.3390/md10102337

**Published:** 2012-10-22

**Authors:** Kazuo Azuma, Toshitsugu Ishihara, Hiroyuki Nakamoto, Takao Amaha, Tomohiro Osaki, Takeshi Tsuka, Tomohiro Imagawa, Saburo Minami, Osamu Takashima, Shinsuke Ifuku, Minoru Morimoto, Hiroyuki Saimoto, Hitoshi Kawamoto, Yoshiharu Okamoto

**Affiliations:** 1 Department of Veterinary Clinical Medicine, School of Veterinary Medicine, Tottori University, 4-101 Koyama-minami, Tottori-shi, Tottori 680-8553, Japan; Email: kazuazu85@yahoo.co.jp (K.A.); toshitsuguishihara@hotmail.co.jp (T.I.); nkmthryk@gmail.com (H.N.); amahatakao@gmail.com (T.A.); tosaki@muses.tottori-u.ac.jp (T.O.); tsuka@muses.tottori-u.ac.jp (T.T.); imagawat@muses.tottori-u.ac.jp (T.I.); minami@muses.tottori-u.ac.jp (S.M.); 2 Scientific Crime Laboratory, Tottori Prefectural Police H. Q., 2-12 Chiyomi, Tottori 680-0911, Japan; Email: chemtaka826@ncn-t.net; 3 The Graduate School of Engineering, Tottori University, 4-101 Koyama-minami, Tottori 680-8552, Japan; Email: sifuku@chem.tottori-u.ac.jp (S.I.); morimoto@chem.tottori-u.ac.jp (M.M.); saimoto@chem.tottori-u.ac.jp (H.S.); 4 Marine Products Kimura Co., LTD., 3307 Watari-cho Sakaiminato-shi, Tottori 684-0072, Japan; Email: kawamoto@mozuku-1ban.jp

**Keywords:** fucoidan, *Cladosiphon okamuranu*, anti-tumor activities, colon-26, mice, functional food

## Abstract

We evaluated the anti-tumor activities of the oral administration of fucoidan extracted from *Cladosiphon okamuranus* using a tumor (colon 26)-bearing mouse model. The materials used included low-molecular-weight fucoidan (LMWF: 6.5–40 kDa), intermediate-molecular-weight fucoidan (IMWF: 110–138 kDa) and high-molecular-weight fucoidan (HMWF: 300–330 kDa). The IMWF group showed significantly suppressed tumor growth. The LMWF and HMWF groups showed significantly increased survival times compared with that observed in the control group (mice fed a fucoidan-free diet). The median survival times in the control, LMWF, IMWF and HMWF groups were 23, 46, 40 and 43 days, respectively. It was also found that oral administration of fucoidan increased the population of natural killer cells in the spleen. Furthermore, from the results of the experiment using Myd-88 knockout mice, it was found that these effects are related to gut immunity. These results suggest that fucoidan is a candidate anti-tumor functional food.

## 1. Introduction

Fucoidan is a complex sulfated polysaccharide found in the cell walls of several types of edible brown algae. The structure and composition of fucoidan vary among different brown seaweed species; however, the compound generally consists primarily of L-fucose and sulfate along with small quantities of D-galactose, D-mannose, D-xylose and uronic acid [1,2,3]. Many reports indicate that fucoidan exhibits various bioactivities, including anti-viral [4], anti-coagulant [5,6], antioxidant [7], anti-inflammatory [5,8] and immunomodulatory effects [9]. 

The anti-tumor activities of fucoidan have also been observed *in vivo* [10,11,12] and *in vitro* [13,14,15]. The mechanisms underlying the anti-tumor activities of fucoidan have been reported to include the induction of apoptosis [14], the suppression of neovascularity [12] and the activation of cell-mediated immunity [16]. In particular, fucoidan increases the activity of natural killer cells *in vivo* [4,16]. Fucoidan promotes the maturation of human monocytes to dendritic cells. Fucoidan also stimulates the release of interleukin (IL)-12 and interferon-γ *in vitro* [17]. 

Previous reports have indicated that fucoidan is a beneficial anti-tumor agent. However, almost all reports describing the anti-tumor effects of fucoidan *in vivo* have evaluated the administration of fucoidan intraperitoneally, intravenously or by regional injection into tumor tissues [10,11,12]. Few reports have investigated the beneficial effects of oral administration of fucoidan in mouse or rat tumor models. Some reports have indicated that the bioactivity of fucoidan is affected by these variables [5]. Differences in the molecular weight of fucoidan influence the anti-neovascular actions of fucoidan [12] and show different effects in murine splenic NK cells [18]. To the best of our knowledge, no reports have described the effects of differences in the molecular weight of fucoidan on tumor growth and survival time in a tumor-bearing mouse model.

In this study, we examined the effects of oral administration and the molecular weight of fucoidan extracted from *Cladosiphon okamuranus* on tumor growth and survival time using a colon 26 tumor-bearing mouse model. 

## 2. Results and Discussion

### 2.1. Effects of Oral Administration of Fucoidan in a Colon 26 Tumor-Bearing Mice Model

#### 2.1.1. Effects of Oral Administration of Fucoidan on Tumor Growth

**Figure 1:**
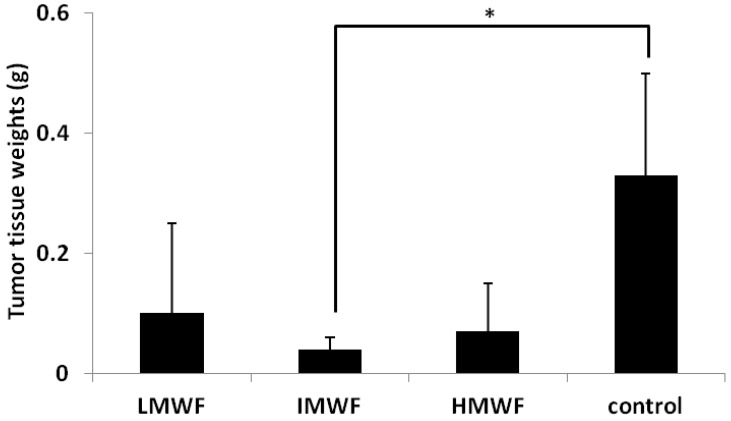
Effects of fucoidan on tumor tissue weights. Date represent the mean ± S.D. *n* = 5 in the low-molecular-weight fucoidan (LMWF), intermediate-molecular-weight fucoidan (IMWF) and high-molecular-weight fucoidan (HMWF) groups, *n* = 10 in the control group. * *p* < 0.05 compared to the control group, Turkey-Kramer’s test.

All group mice fed fucoidan were fed about 5 g/kg·day of each fucoidan. Following the oral administration of fucoidan in a colon 26 tumor-bearing mice model, a comparison of tumor tissue weight according to the molecular weight of fucoidan was made, the results of which are shown in Figure 1. The tumor tissue weights were significantly decreased in the intermediate-molecular-weight fucoidan (IMWF: 110–138 kDa) group compared to those observed in the control group (the mice fed a fucoidan-free diet) (control group: 0.33 ± 0.17 g, IMWF group: 0.05 ± 0.03 g, *p* < 0.05). The tumor weights were decreased in both the low-molecular-weight fucoidan (LMWF: 6.5–40 kDa) and high-molecular-weight fucoidan (HMWF: 300–330 kDa) groups (LMWF group: 0.12 ± 0.16 g, HMWF group: 0.08 ± 0.09 g). However, no significant changes were observed compared with that seen in the control group.

We examined the effects of IMWF on histopathological changes in tumor tissue using hematoxylin-eosin (HE) staining. In the IMWF and control groups, many cell divisions were observed. The number of cell divisions in each group is shown in Table 1. The number of cell divisions in the IMWF group was significantly decreased compared to that observed in the control group. We also examined the effects of IMWF on apoptosis using terminal deoxynucleotidyl transferase-mediated deoxyuridine triphosphatate-biotin nick end labeling (TUNEL) staining. TUNEL-positive cells were observed occasionally in both the IMWF and control groups. The numbers of TUNEL-positive cells in the IMWF and control groups are shown in Table 1. The number of TUNEL-positive cells in the IMWF group was increased slightly compared to that observed in the control group.

**Table 1:** Effects of fucoidan on the numbers of cell division cells and triphosphatate-biotin nick end labeling (TUNEL) positive cells in tumors. Data are represented as mean ± S.D. ** *p* < 0.01 compared to the control group.

	IMWF	Control
Cell division cells (cells/field)	93.3 ± 20.4 **	151.7 ± 14.5
TUNEL positive cells (cells/field)	33.0 ± 12.8	24.3 ± 4.7

#### 2.1.2. Effects of Oral Administration of Fucoidan on Survival Times

**Figure 2:**
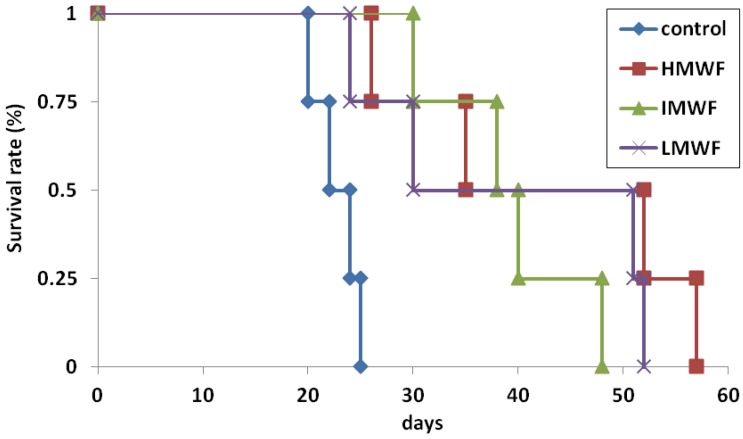
Effects of oral administration of fucoidan on survival times. *n* = 4 in each group. *p* < 0.05 compared to the control group in the LMWF and HMWF groups by Longrank test.

The effects of fucoidan on the survival time in colon 26 tumor-bearing mice are shown in Figure 2. The survival times of the mice in the LMWF, IMWF and HMWF groups were prolonged. The survival times of the mice in the LMWF and HMWF groups were significantly increased compared to that observed in the control group (log-rank test, *p* < 0.05). The median survival time was 23 days in the control group, 46 days in the LMWF group, 40 days in the IMWF group and 43 days in the HMWF group. 

Previous reports have indicated that fucoidan exhibits anti-tumor actions via several mechanisms. Fucoidan exhibits anti-neovascular effects [12]. Fucoidan also suppresses the adhesion of tumor cells to normal tissues. Liu *et al.* reported that fucoidan inhibits the adhesion of MDA-MB-231 cells to fibronectin [19]. Fucoidan also induces apoptosis in tumor tissue [14,20]. In human colon cancer cells, apoptosis induced by fucoidan is mediated via both death receptor-mediated and mitochondria-mediated apoptotic pathways [20]. In our results, fucoidan slightly increased the number of TUNEL-positive cells *in vivo*. The suppressive effects of fucoidan on tumor growth might arise from these mechanisms. Aisa *et al.* reported that fucoidan inhibits cell cycles depending on the concentration of fucoidan and the culture duration [14]. Additionally, fucoidan increases the number of interkinesis cells. In this study, fucoidan significantly decreased the number of mitotic cells *in vivo*. Our results indicate that fucoidan modulates cell cycles.

Our results indicate that differences in the molecular weight of fucoidan influence fucoidan’s anti-tumor effects *in vivo*. Fucoidan is not degraded by human digestive enzymes [21]. We examined the absorption of fucoidan in murine small intestines. The results showed that no amount of fucoidan was absorbed in murine small intestines (data not shown). These phenomena indicate that the bioactivity of fucoidan that occurs *in vivo* is not related to absorption from the gut. The anti-tumor activity of fucoidan observed *in vivo* might be a result of indirect actions such as stimulation of the gut immune system. Our results indicate that stimulation of the gut immune system by fucoidan *in vivo *depends on fucoidan’s molecular weight. 

### 2.2. Effects of Oral Administration of Fucoidan on Splenic NK Cell Populations

**Figure 3:**
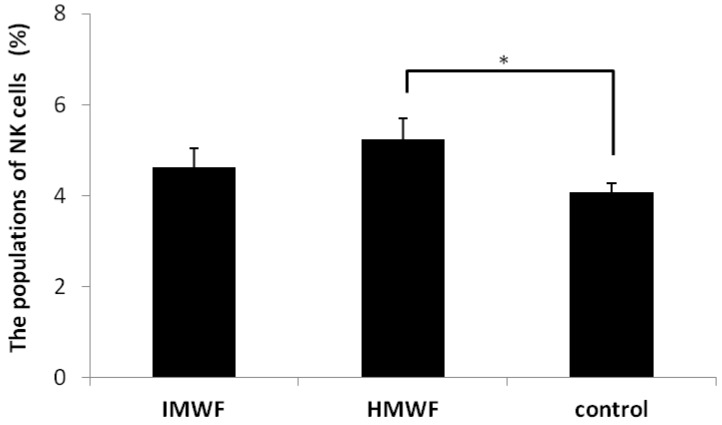
The effects of oral administration of fucoidan on murine splenic natural killer (NK) cell populations. The data represent the mean ± S.D. *n* = 3 of each group. * *p* < 0.05 compared to the control group, Tukey-Kramer’s test.

The effects of oral administration of IMWE and HMWF on murine splenic NK cell populations are shown in Figure 3. Oral administration of IMWF (4.6 ± 0.4%) and HMWF (5.2 ± 0.5%) increased the size of the NK cell population in splenic tissue compared with that observed in the control group (4.1 ± 0.2%). There was a significant difference between the HMWF group and the control group. 

Some reports have indicated that fucoidan stimulates NK cell activity *in vivo* [16,22,23]. Previous reports have also shown that fucoidan extracted from *Cladosiphon okamuranus* increases the size of NK cell populations. In this study, the population of NK cells was increased significantly in the HMWF group compared to that observed in the control group. Shimizu *et al*. reported that high-molecular-weight fucoidan extracted from *Cladosiphon okamuranus* (200–300 kDa) promotes greater increases in the proportion of murine cytotoxic T cells than middle- (2–3 kDa) or low- (0.5–1 kDa) molecular-weight fucoidan [18]. They administered fucoidan by containing 5% fucoidan in laboratory chew for 70 days. In the present study, we also administered the same amount of fucoidan for 70 days. Our results indicate feedings fucoidan also increase the population of the NK cells in mouse spleen. NK cells have the ability to recognize and destroy a wide range of abnormal cells, including tumor cells, without damaging healthy and normal self cells [24]. *In vivo* and *in vitro* studies have shown that NK cells can eliminate tumor cells [25]. Our results indicate that one of the anti-tumor mechanisms of fucoidan extracted from *Cladosiphon okamuranus* might be to mediate increases in NK cell activity. Our results also indicate that differences in the molecular weight of fucoidan might affect NK cell activity *in vivo*. Further studies must be conducted in order to understand the relationships between the molecular weight of fucoidan and NK cell activity *in vivo*. 

### 2.3. Effects of Oral Administration of Fucoidan on Tumor Growing and Serum Cytokine in Myd-88 Knockout Mice

To reveal the relationships between the anti-tumor effects of fucoidan and innate immunity, we examined the anti-tumor effects of fucoidan using myeloid differentiation primary response gene-88 (Myd-88) knockout mice. The results are shown in Table 2. No significant changes in tumor weight were observed in the HMWF/Myd-88 group compared to that seen in the control/Myd-88 group. No change was observed in the serum IL-2 level between the HMWF/Myd-88 and control/Myd-88 groups. The serum IL-10 level in the HMWF/Myd-88 group was increased slightly compared to that observed in the control/Myd-88 group.

**Table 2:** Effect of oral administration of fucoidan on tumor growth and serum cytokine level in Myd-88 knockout mice. Data represent mean ± S.D. *n* = 5 in each group. * *p * < 0.05 compared to the control/Myd-88 group, Welch’s *t*-test.

	HMWF/Myd-88 group	control/Myd-88 group
tumor weight (g)	0.15 ± 0.06	0.18 ± 0.06
serum IL-2 (pg/mL)	1.03 ± 0.01	1.00 ± 0.02
serum IL-10 (pg/mL)	2.93 ± 1.23	2.12 ± 1.59

Previous reports have indicated that the bioactivity of fucoidan activates innate immunity [4,16,17]. The activation of innate immunity is essential for the activation of adaptive immunity [26]. In particular, toll-like receptors (TLR) on the surface of intracellular organelles recognize the specific structures of bacteria, viruses and fungi [27]. Adapter molecules such as Myd-88 and TIR-domain-containing adapter-inducing interferon-β (TRIF) play important roles in inducing the production of cytokines via TLRs [28,29]. In our results, fucoidan exhibited no anti-tumor effects in Myd-88 knockout mice. This result indicates that the anti-tumor effects of fucoidan arise from the Myd-88 pathway *in vivo*.

The serum IL-2 level in the HMWF/Myd-88 group was not changed compared to that observed in the control/Myd-88 group. The serum IL-10 level in the HMWF/Myd-88 group was increased compared to that observed in the control/Myd-88 group. TLR-4 stimulates cytokine production via both Myd-88 and TRIF signaling pathways [29]. Our results indicate that increased serum cytokine levels might result from the TRIF-dependent pathway, not the Myd-88-dependent pathway. To our knowledge, there are no reports investigating the relationships between TLRs and fucoidan. Our results indicate that fucoidan stimulates TLR-4 in tumor mouse models. To understand the mechanisms underlying the anti-tumor effects of fucoidan, further studies focusing on TLRs must be conducted both *in vivo* and *in vitro*.

Some reports have indicated that the bioactivity of fucoidan depends on fucoidan’s molecular weight [18,30,31]. Yang *et al.* described the anti-cancer activities of fucoidan extracted from *Undaria pinnatifida*. Their results indicated that 490 kDa of fucoidan exhibits more effective anti-cancer activities than natural fucoidan (5100 kDa) or low-molecular-weight fucoidan (260 kDa) [30]. Shimizu *et al*. reported that high-molecular-weight fucoidan promotes greater increases in the proportion of murine cytotoxic T cells than middle- or low- molecular-weight fucoidan [18]. Park *et al.* reported that high-molecular-weight fucoidan enhances inflammation, while low-molecular-weight fucoidan shows anti-inflammatory activities through the suppression of Th1-mediated immune reactions [31]. Our results demonstrated the presence of HMWF anti-tumor activities following activation of innate immunity *in vivo*. To understand the mechanisms of the anti-tumor activities exhibited by IMWF and LMWF, further studies must be conducted. 

In this study, we used fucoidan which was not changed the composition and sulfation degree in the process of hydrothermal treatment. Bilan *et al*. reported even a single algal species might contain, at least in minor amounts, several sulfated polysaccharides differing in molecular structure [32]. This result indicates fractionation of crude “fucoidans” may give non-fucoidan polysaccharides. Some results indicate the bioactivities of fucoidan are depended on not only their molecular weight but also their derived seaweeds and compositions [5,6]. When we compare the bioactivities of polysaccharides such as fucoidan, not only the molecular weight but also the derived seaweeds and compositions must be considered.

## 3. Experimental Section

### 3.1. Animals

BALB/c mice (4- or 5-week-old, female) were purchased from CREA Japan (Osaka, Japan). Myd-88 knockout mice (BALB/c background, 4-week-old, female) were purchased from Oriental BioService, Inc., (Kyoto, Japan). All mice were maintained under conventional conditions. The use of the mice and the procedures used in this study were approved by the Animal Research Committee of Tottori University.

### 3.2. Reagents

Fucoidan (HMWF group; *M*_w_ 300–330 kDa, lot No.1010) from *Cladosiphon okamuranus* was provided by Marine Products Kimuraya Inc. (Tottori, Japan). Teruya *et al*. reported that fucoidan from *Cladosiphon okamuranus* consisted of L-fucose, D-xylose, D-gulucuronic acid in the ratio of 4.0:0.03:1.0 [33]. Degree of sulfation (DS) was evaluated by the elemental analysis method to be 0.33–0.34. Degree of acetylation (DA) was determined by the ^1^H NMR analysis method to be 0.2. Fucoidan samples of various molecular weights were prepared by the application of hydrothermal depolymerization method [34]. These values are given per mole of fucose residues. The molecular weights (MW) of fucoidan were calculated by the gel permeation chromatography method (columns: TSKgel PW_XL_ (Tosoh CO., Tokyo, Japan); mobile phase: 0.1 M NaNO_3_; flow rate: 0.5 mL/min; column temperature: 40 °C; detector: RI; calibration: pullulan standard). Hydrothermal treatment of aqueous solution of fucoidan at 140 °C for 15–60 min gave the low molecular weight fucoidan group (LMWF group; *M*_w_ 6.5–40 kDa). Similar treatment at 140 °C for 5–8 min gave intermediate molecular weight fucoidan group (IMWF group; *M*_w_ 110–138 kDa). Under these hydrothermal conditions, all samples maintained their DS and DA values.

### 3.3. Preparation of the Tumor-Bearing Mouse Model

The murine colon cancer cell line (colon 26) was provided in 2-mm tumor tissue blocks by Cancer Institute Hospital (Tokyo, Japan). The blocks were dipped into cell banker^®^ (ZENOAQ, Inc., Fukushima, Japan) and stored at −80 °C until use.

The mice were anesthetized with inhalation of 3%–5% isoflurane (Intervet, Inc., Tokyo, Japan). The tumor tissue blocks were thawed at room temperature and washed three to four times with saline. The tumor tissue blocks were minced to 1-mm blocks, and pieces of the blocks were transplanted subcutaneously into the dorsal regions of the mice.

When the transplanted tumors grew to 10 mm in size, the mice were euthanized with inhalation of 5% isoflurane followed by cervical dislocation. The tumor tissues were extirpated, and the coats, microvessels and necrotic tissues were removed. After being washed with saline, the tumor tissues were minced to 1-mm blocks and transplanted subcutaneously into the dorsal regions of the mice. We used tumor tissues with passage numbers greater than two in our experiments.

### 3.4. Tumor Growth Study

Twenty-five BALB/c mice were randomized into four groups: the control group (control, *n* = 10), the LMWF group (LMWF, *n* = 5), the IMWF group (IMWF, *n* = 5) and the HMWF group (HMWF, *n* = 5).

Except for the control group, each fucoidan group was fed 5% (w/w) fucoidan with a normal powdered diet (CE-2; CREA Japan, Osaka, Japan). All group mice fed fucoidan were fed about 5 g/kg·day of fucoidan. The control group was fed a normal powdered diet. Before transplantation of the tumor tissues, the mice were bred for 28 days by being fed a powdered diet with or without each weight of fucoidan. After being bred for 28 days, each mouse underwent subcutaneous transplantation of one piece of tumor tissue into the dorsal region. Each mouse had been fed a powdered diet with or without fucoidan for 14 days. Then, all mice were euthanized with inhalation of 5% isoflurane followed by cervical dislocation. The tumor tissues were removed and weighed (g). After being weighed, the tumor tissues were fixed in 10% buffered formalin. 

### 3.5. Histopathological Evaluation

In the control and IMWF groups, thin sections (5 μm) were made from each sample for histopathological observation after hematoxylin-eosin (HE) staining. Each section was examined microscopically, and the number of cell divisions in each tumor was calculated. The calculation of cell divisions was performed in 10 fields at 400× magnification using five mice in each group. The mean score of 50 fields was considered to be the number of cell divisions in each group.

We also examined the apoptosis rate of the tumor tissues using terminal deoxynucleotidyl transferase-mediated deoxyuridine triphosphatate-biotin nick end labeling (TUNEL) staining. Each section was examined microscopically and the number of cell divisions in each tumor was calculated. The calculation of cell divisions was performed in 10 fields at 400× magnification using four mice in each group. The mean score of 40 fields was considered to be the number of cell divisions in each group.

### 3.6. Survival Time Study

Twenty-four BALB/c mice were randomized into four groups: the control group (control, *n* = 4), the LMWF group (LMWF, *n* = 4), the IMWF group (IMWF, *n* = 4) and the HMWF group (HMWF, *n* = 4).

Except for the control group, each fucoidan group was fed 5% (w/w) fucoidan with a normal powdered diet (CE-2; CREA Japan, Osaka, Japan). The control group was fed a normal powdered diet. Before transplantation of the tumor tissues, the mice were bred for 28 days by being fed a powdered diet with or without each weight of fucoidan. After being bred for 28 days, each mouse underwent subcutaneous transplantation of one piece of tumor tissue into the dorsal region. After the tumor tissues were transplanted (day 0), the number of days until the mice died was measured. Thereafter, a survival curve was created and the median survival time (days) was calculated.

### 3.7. Measurements of Splenic Natural Killer T Cells (NK Cells)

Nine BALB/c mice were randomized into three groups: the control group (control, *n* = 3) fed a normal powdered diet (CE-2, CREA Japan, Osaka, Japan) for 70 days, the IMWF group (IMWF, *n* = 3) fed a normal powdered diet with 5% (w/w) IMWF for 70 days and the HMWF group (HMWF, *n* = 3) fed a normal powdered diet with 5% (w/w) HMWF for 70 days.

After breeding, all mice were euthanized with inhalation of 5% isoflurane followed by cervical dislocation. Spleens were obtained from each mouse and splenocytes were collected. Erythrocytes were removed by mixing with a lysis buffer (150 mM NH_4_Cl, 10 nM KHCO_3_ and 100 μM EDTA) for 10 min at room temperature. 

FITC-conjugated anti-mouse NKG2A/C/E monoclonal antibodies (BD Bioscience, San Jose, CA) and PE-conjugated anti-mouse CD8a monoclonal antibodies (BD bioscience, San Jose, CA) were used. FITC- and PE-conjugated IgG2a isotype-matched antibodies were used as controls for background staining. Leukocytes were then stained at 4 °C using predetermined optimal concentrations of each antibody for 30 min. Antibody binding was analyzed on a FACSAria flow cytometer (BD Biosciences) by gating the cells with the forward and side light scatter properties of lymphocytes. NKG2A/C/E-positive and CD8a-negative lymphocytes were deemed to be NK cells. We calculated the percentage of NK cells in the splenic lymphocytes.

### 3.8. MyD-88 Knockout Mice Study

Ten Myd-88 knockout mice were divided into two groups (*n* = 5 each): the control/Myd-88 (control, *n* = 5) and the HMWF/Myd-88 groups. The HMWF/Myd-88 group was fed 5% (w/w) fucoidan with a normal powdered diet (CE-2; CREA Japan, Osaka, Japan). The control/Myd-88 group was fed a normal powdered diet. Before transplantation of the tumor tissues, the mice were bred for 28 days by being fed a powdered diet with or without fucoidan. After being bred for 28 days, each mouse underwent subcutaneous transplantation of one piece of tumor tissue into the dorsal region. Each mouse had been fed a powdered diet with or without fucoidan for 14 days. Then, all mice were euthanized with inhalation of 5% isoflurane followed by cervical dislocation. Tumor tissues and sera were obtained. The tumor tissues were weighed. 

The serum interleukin (IL)-2 and IL-10 levels were quantified using a sandwich enzyme-linked immunosorbent assay (ELISA). The serum IL-2 and IL-10 levels were measured using murine IL-2 and IL-10 ELISA kits (Thermo SCIENTIFIC, Rockford, IL, USA) according to the manufacturer’s protocols.

### 3.9. Statistical Analysis

The data are expressed as the mean ± S.D. The statistical analyses were performed using one-way ANOVA followed by Tukey-Kramer’s test or Welch’s *t*-test. We performed the log-rank test to evaluate the survival times of the tumor mice. A *p*-value < 0.05 was considered statistically significant.

## 4. Conclusions

In this study, we examined the effects of oral administration of fucoidan extracted from *Cladosiphon okamuranus* on tumor growth and survival time in a colon 26 tumor-bearing mouse model. Our results indicate that oral administration of fucoidan suppresses tumor growth and prolongs survival times in colon 26 tumor-bearing mice. Our results also indicate that the molecular weight of fucoidan might be related to fucoidan’s anti-tumor effects. 

Furthermore, the results indicate that the underlying anti-tumor mechanisms of fucoidan might include increasing the size of the population of NK cells and stimulating gut immunity. These results suggest that fucoidan is a beneficial functional food for cancer patients.
